# Supplementary material for: Polyketide synthase gene clusters in the ericoid mycorrhizal fungus Oidiodendron maius: genome-wide prediction, expression profiling, and functional characterization of knockout mutants
Source: Front Plant Sci. 2026 Sep 16;17:1924430. doi: 10.3389/fpls.2026.1924430 (PMC13623903; doi:10.3389/fpls.2026.1924430)
Supplement: Supplementary file 1 [file SupplementaryFile1.zip › Supplementary Materials/Supplementary Folder 1.zip!/Oidma1_AssemblyScaffolds_Repeatmasked/knownclusterblast/cluster45/jgi.p_Oidma1_45633_mibig_hits.html]

| MIBiG Protein | Description | MIBiG Cluster | MiBiG Product | % ID | % Coverage | BLAST Score | E-value |
| --- | --- | --- | --- | --- | --- | --- | --- |
| ABV91295.1 | putative\_multidrug\_transporter | BGC0000158 | Tautomycetin\_biosynthetic\_gene\_cluster | 29 | 77.094972067 | 166 | 8.3e-41 |
| AFU65888.1 | DacR2 | BGC0000216 | Dactylocycline\_biosynthetic\_gene\_cluster | 28 | 75.4189944134 | 146 | 6.8e-35 |
| ABB05093.1 | LipEx1 | BGC0001003 | Lipomycin\_biosynthetic\_gene\_cluster | 23 | 79.7020484171 | 90 | 7.5e-18 |
